# Whole-genome sequencing of five culturable endophytes isolated from garlic flower stem sap from Upstate New York, USA

**DOI:** 10.1128/mra.01157-24

**Published:** 2025-03-13

**Authors:** Girish Kumar, Han Ming Gan, André O. Hudson, Michael A. Savka

**Affiliations:** 1 Program in Biotechnology and Molecular Bioscience, The Thomas H. Gosnell School of Life Sciences, Rochester Institute of Technology 173222 , Rochester, New York, USA; 2 Patriot Biotech Sdn Bhd, Subang Jaya, Malaysia; The University of Arizona, Tucson, Arizona, USA

**Keywords:** *Allium sativum*, bacterial endophytes, garlic sap endophytes, genomes

## Abstract

We report the whole-genome sequences of five culturable bacterial endophytes isolated from German hardneck garlic sap (*Allium sativum* L.). Three isolates were classified under the genus *Sphingomonas*, while the others were identified as *Staphylococcus warneri* and *Frigoribacterium faeni*. This data set will be helpful in enhancing garlic cultivation practices.

## ANNOUNCEMENT

Endophytes are a group of microorganisms that live inside plants without causing visible harm (1). Recent research has shown that endophytes interact with plants more effectively than rhizospheric bacteria (2, 3). In a previous study, we performed the whole-genome sequencing (WGS) of five culturable bacterial endophytes associated with garlic cloves (4). In this study, we are presenting the WGS of five culturable bacterial endophytes that are associated with the endogenous sap of the garlic plant.

Mature field-grown hardneck German garlic bulbs were obtained from Bischoping Farm, Rush New York (Longitude: −77.6374368; Latitude: 43.01495449999999) in August 2022. Garlic plants with flower stem were decapitated aseptically with a sterile scalpel, and the stem sap that accumulated on the cut surface was aseptically harvested using a micropipette with sterile tips. The harvested sap was then transferred to sterile Eppendorf tubes. The sap was then placed into tryptic soy broth (TS) medium and incubated at 28°C for 3 days for bacterial isolation. The resulting culture was plated onto TS agar media and incubated under the same conditions to isolate culturable sap endophytes. Following this, five distinct colonies with unique morphologies were obtained and subcultured on TS agar medium at 28°C for 48 hours to confirm purity.

The genomic DNA was extracted from 25 mg of cell culture using the E.Z.N.A. bacterial DNA kit (Omega Bio-Tek, Norcross, GA). The DNA concentrations were measured using a Qubit 3.0 fluorometer (1× dsDNA High Sensitivity Kit; Life Technologies, MD). Next, sequencing libraries were prepared according to the Nextera XT library preparation protocols and sequenced on the MiSeq (Illumina, San Diego, CA) with a MiSeq reagent V3 kit (2 × 300 cycles) at the Genomics Lab, Rochester Institute of Technology, following the manufacturer’s guidelines. Raw reads were then processed for quality control using the fastp program version 0.23.2 (5) and assembled into contigs using the SPAdes genome assembler (6; version 3.15). The NCBI Prokaryotic Genome Annotation Pipeline (7; version 6.5 [http://www.ncbi.nlm.nih.gov/genome/annotation_prok/]) was used to automatically annotate the genomes upon upload, with default parameters used for all software unless otherwise specified.

Among the five sequenced endophytes, three were identified under genus *Sphingomonas*. This genus is known for its metabolic versatility and common association with plants, as it can utilize various natural compounds (8) and certain environmental contaminants (9). The remaining two endophytes were identified as *Staphylococcus warneri* and *Frigoribacterium faeni*. Although *S. warneri* is typically associated with humans, it has been recently reported to be an unconventional plant pathogen associated with canker disease (10). On the contrary, *Frigoribacterium* has been demonstrated to possess plant growth promotion capabilities (11). The whole-genome sequencing data presented in this study can aid in the identification of genes associated with endophyte-mediated resistance and pathogenicity in garlic, thereby contributing to the improvement of garlic cultivation practices.

**TABLE 1:** Genome annotation information for the five German hardneck garlic sap-associated bacteria endophytes

Sample	# of raw reads	# of nucleotides sequenced	SRA accession	Assembly accession	Taxonomic designation (NCBI)	gANI	Assembly size (bp)	Coverage (X)	# contigs	N50	Gc (%)	# Genes
RIT-Gs-01	2,037,190	477909638	SRR22751342	GCA_029623395.1	Sphingomonas echinoides	83.30	4,391,097	114	95	108462	65	4121
RIT-Gs-02	1,666,648	347263619	SRR22751341	GCA_029623335.1	Staphylococcus warneri	99.64	2,512,126	140	53	95279	32.51	2485
RIT-Gs-03	2,214,878	513286309	SRR22751340	GCA_029623415.1	Sphingomonas faeni	89.62	4,232,894	109	54	166312	64.94	3907
RIT-Gs-04	2,010,452	459997743	SRR22751339	GCA_029623345.1	Sphingomonas phyllosphaerae	92.02	4,319,995	121	55	172865	69.17	4017
RIT-Gs-05	1,927,924	488115597	SRR22751338	GCA_029623355.1	Frigoribacterium faeni	85.00	3,290,706	148	212	26473	71.5	3146

## Data Availability

The accession numbers for each SRA and genome assembly can be found in Table 1. The isolates are not deposited in a culture collection but are maintained in the corresponding author's bacterial freezer stocks and can be provided upon request.
